# Symptomatic Intrathecal Hematoma following an Epidural Blood Patch for an Obstetric Patient with Postdural Puncture Headache: A Case Report and Synthesis of the Literature

**DOI:** 10.1155/2020/8925731

**Published:** 2020-03-18

**Authors:** Hailey J. McInerney, Manuel Lee, Tracie Saunders, Joy Schabel, Rishimani S. N. Adsumelli

**Affiliations:** ^1^Renaissance School of Medicine at Stony Brook University, Stony Brook, NY 11794, USA; ^2^Department of Anesthesia, Renaissance School of Medicine at Stony Brook University, Stony Brook, NY 11794, USA

## Abstract

Epidural blood patch (EBP), generally considered a low-risk procedure, can potentially lead to significant neurological complications. We report the case of a parturient who underwent an uneventful EBP for postdural puncture headache (PDPH) and subsequently presented with progressively worsening radicular symptoms. Magnetic resonance imaging (MRI) revealed an intrathecal hematoma, and conservative management with steroids led to complete recovery. Our case highlights the possibility of this rare complication following an uneventful procedure and the importance of prompt diagnosis and treatment to prevent serious adverse outcomes. Literature review, EBP alternatives, and strategies to minimize complications following blood patch will be discussed in this report.

## 1. Introduction

Epidural blood patch (EBP) remains the treatment of choice for postdural puncture headache (PDPH) when conservative management is unsuccessful or not feasible. The EBP was first described in 1960 by James Gormley, a general surgeon, who injected two to three milliliters of fresh autologous blood into the presumed epidural space of patients who had PDPH after spinal anesthesia. Gormley reported a 100% success rate in seven cases [1]. Since then, EBP has become the gold standard for treating PDPH. Adverse effects of EBP are typically mild and temporary. However, serious complications such as radiculopathy, infection, subdural, and intrathecal hematomas have also been reported in the literature.

We report the case of a 30-year-old parturient with progressively worsening low back pain and radicular symptoms six days following an uneventful EBP. The EBP was performed to treat PDPH following an accidental dural puncture with a Tuohy needle during labor epidural placement. Lumbar spine magnetic resonance imaging (MRI) revealed an intrathecal hematoma in the lumbosacral area. Conservative management led to the eventual resolution of her radicular symptoms. Our case highlights the possibility of this rare complication following an uneventful EBP. We obtained written consent to report this patient's health information.

## 2. Case Description

A healthy parturient with no significant medical history and body mass index (BMI) of 22.86 requested epidural analgesia during her labor. First attempt with a 17-gauge Tuohy needle at the L3-L4 interspace resulted in an accidental dural puncture. The second attempt at the L2-L3 interspace was successful. Four hours after the epidural catheter placement, the patient delivered a healthy baby, and the epidural catheter was removed from the patient later that day.

The patient was noted to have a postdural puncture headache (PDPH) on postpartum day (PPD) 1. The patient opted for an epidural blood patch (EBP) after risks, benefits, and alternatives were explained. The EBP was performed by an experienced obstetric anesthesiologist. A 17-gauge Tuohy needle was inserted into the presumed L3-L4 interspace, and the epidural space was confirmed with loss of resistance with air technique. Autologous blood was injected slowly and stopped when the patient complained of back pressure. A total of 21 milliliters of autologous blood were injected. The patient reported immediate relief of her headache. On PPD 2, the patient reported mild backache but complete resolution of her headache. She was discharged home and reported only mild backache on PPD 3 and 4 during phone follow-up.

**Figure 1:**
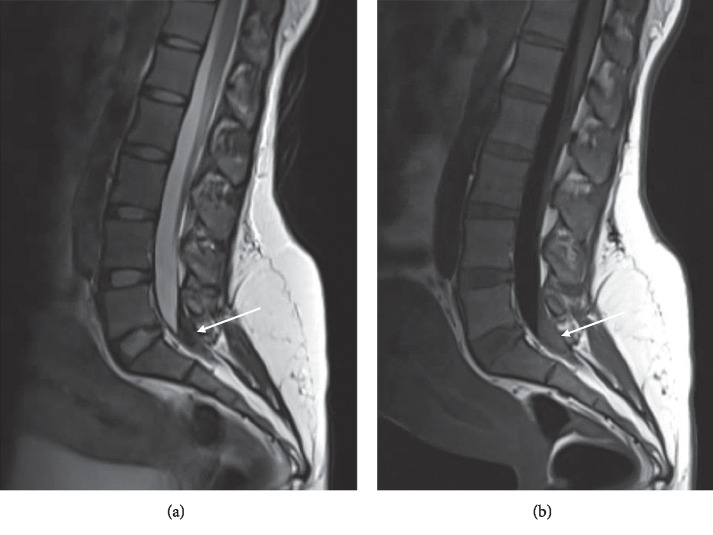
Sagittal T2-weighted Dixon fat saturated (a) and T1-weighted FLAIR (b) images showing blood collection within the thecal sac extending from the level of the L4-L5 decubitus base down to the level of S1-S2. This is most pronounced at the level of L5-S1 where the hematoma occupies the entire thecal sac and surrounds the cauda equina (arrow).

On PPD 6, the patient presented to Labor and Delivery with a chief complaint of severe back and bilateral leg pain. She reported low back pain with radiation to her abdomen and down her legs. She described the pain as “electric shocks” and “shooting pain” which was worse with movement and the supine position. She had no motor weakness or bladder and bowel dysfunction. MRI of the lumbar spine revealed hemorrhage within the thecal sac extending from the L4-L5 intervertebral disc space down to level S1-S2, most significantly at L5-S1 where the hemorrhage filled the entire thecal sac and surrounded the cauda equina (Figure 1). Neurosurgery recommended dexamethasone (two milligrams intravenously every six hours), neurologic checks every two hours, and monitoring for changes in leg strength, bowel or bladder incontinence, and saddle anesthesia. The patient was admitted for medical treatment and monitoring. By PPD 8, the patient was ambulating with some mild pain in her hips but reported significant improvement in her symptoms and was discharged. By PPD 16, her pain steadily improved with only mild pain when sitting, and she made full recovery.

## 3. Discussion

To our knowledge, there exist only eight other reported cases of an intrathecal or subdural hematoma in the setting of EBP placement (Table 1). All patients described in these cases were treated with conservative management, and in the majority of cases, the symptoms resolved. The exact mechanism of intrathecal hematoma development is unknown, but several hypotheses must be considered.

The first hypothesis is that the blood was deposited into the epidural compartment as intended; however, the increase in pressure so close to the large communication made by the original puncture with the 17-gauge Tuohy needle might have pushed some blood into the subdural or intrathecal compartments [2]. Beards et al. examined 5 patients with MRI at 30 min, 3 h, and 18 h after blood patching and demonstrated that the bulk of the spread in the epidural space extended three to five vertebral levels with a tendency toward cephalad spread. This suggests that perhaps performing the EBP at one to two levels caudal to the original dural puncture site may be just as efficacious and could reduce the possibility of blood being pushed into another compartment. Of particular interest here, Beards did note the spread of blood into the subarachnoid space in two patients, which he presumed extended there through the puncture site.

A second hypothesis is that the blood was injected into either the subdural space with subsequent leak into the intrathecal compartment, or that it was injected directly into the subarachnoid space. A case of spinal subdural hematoma following EBP by Verduzco et al. [3] supports this possibility as the lumbar spine MRI did not show evidence of blood or fluid in the epidural space (Table 1). The authors hypothesized that the Tuohy needle was placed subdural during the EBP and that increased pressure from injection of blood caused dissection of the dura and arachnoid mater. The safety and success of the EBP hinges on accurately injecting the blood into the intended space. However, the loss of resistance (LOR) technique, the current standard for identifying needle-tip position in the epidural space, is prone to both false positive and false negative errors. Bartynski et al. described the LOR technique as an inadequate method for identifying the epidural space that results in incorrect placement at the high rate of 25.7% in patients undergoing lumbar epidural steroid injection [4]. The authors concluded that a fluoroscopic epidurogram or computed tomography (CT) guidance is essential for correct identification of needle-tip placement in the epidural space.

Taken altogether, these cases highlight the need for prompt evaluation with lumbar spine MRI when a patient presents with severe back pain or lower extremity radicular symptoms following EBP. A multidisciplinary approach should involve consultation with neurology and neurosurgery to determine the most appropriate treatment options. MRI is an important tool which may be used not only to confirm the diagnosis, but also to monitor the recovery process [5].

 Given the possible complications of EBP placement, it is important to consider alternative practices to minimize the need for EBP. One practice strategy is to insert an intrathecal catheter during the inadvertent dural puncture, which has been shown to significantly reduce the risk for EBP by decreasing the severity of the headache [6]. Another strategy to minimize the need for EBP would be to perform either a sphenopalatine ganglion block or a suboccipital muscle injection. A bilateral transnasal sphenopalatine block has been shown to be a simple, effective, and minimally invasive way to manage moderate PDPH [7]. Alternatively, an ultrasound-guided injection of a dexamethasone-lidocaine mixture in the suboccipital muscles has also been shown to be effective in managing PDPH [8]. Lastly, there have been several pharmacologic approaches reported to be effective in the management of PDPH including odansetron, intravenous aminophylline, epidural morphine, and intravenous cosyntropin [9]. Caffeine has shown effectiveness for treating PDPH, and gabapentin and hydrocortisone have been reported to decrease pain severity scores [10].

If an EBP is performed, it may be prudent to avoid the same interspace as the original dural puncture. Blood injected with the Tuohy needle at the original dural puncture site has the potential to spread to the subdural and intrathecal space [2]. Case reports that mention the interspace level of blood injection also support this possibility [11, 12]. Moving forward, using fluoroscopic guidance to confirm needle-tip position and the spread of injected blood during EBP placement may be useful and feasible in large academic centers such as ours.

In addition to implementing strategies both to accurately identify the epidural space during EBP placement and to minimize the need for EBP placement, all patients treated for PDPH should be carefully followed after discharge. Consistent patient follow-up would allow prompt awareness and treatment of worsening or new symptoms and improve patient outcomes.

## Figures and Tables

**Table 1:** Previously reported obstetric cases which resulted in intrathecal or subdural hematoma following epidural blood patch.

Study	Initial procedure	Blood patch	Symptoms	MRI findings	Outcome
Kalina et al. [5]	Epidural catheter for labor analgesia, unspecified interspace	27 mL, unspecified interspace, PPD 4	Progressive severe back pain and radicular symptoms, over the following several days	Intrathecal hematoma	Symptoms gradually improved over several months

Verduzco et al. [3]	Spinal anesthesia for postpartum tubal ligation, L3-L4 interspace	20 mL, L3-L4 interspace, 12 h post-op	Throbbing pain in waist and bilateral buttocks with radiation to lateral aspect of both thighs, POD 10	Multiple subacute hemorrhagic collections in the posterior cauda equina within the subdural space from L1 to S3. No epidural blood or fluid collection	Complete resolution of symptoms by 5 weeks post-op

Devroe et al. [13]	Epidural catheter for labor analgesia, unspecified interspace	20 mL, same interspace as dural puncture, PPD 4	Back pain with lumbar muscle spasms radiating to both buttocks and legs with difficulties passing urine, PPD 5	Large subdural hematoma from T8 to L5 with compression of the spinal medulla	Symptoms improved over 4 days and MRI two weeks later indicated almost complete resolution of subdural hematoma

Hudman et al. [14]	Epidural catheter for labor analgesia, unspecified interspace	Unspecified volume and interspace, PPD 2, PPD 5	Lower back pain that radiated to left leg, PPD 10	Intrathecal hematoma from L5 to sacral canal	Complete resolution of symptoms PPD 20

Roy–Gash et al. [11]	Epidural catheter for labor analgesia, L3-L4 interspace	30 mL, L3-L4 interspace, PPD 3	Neck stiffness, fever, intense lower back pain, bilateral leg pain, PPD 7	Intrathecal hematoma from L5 to end of dural sac	Complete resolution of neurological symptoms, 4 weeks postpartum

Kearsley et al. [15]	Spinal anesthesia for cesarean section, unspecified interspace	20 mL, L5-S1, POD 3 26 mL, L5-S1, POD 8	Severe back pain with radiation to lower limbs, “electricity-like” sensation, POD 15	Subdural hemorrhage with mass effect	Complete resolution of symptoms and MRI findings, POD 32

Iga et al. [12]	Combined spinal epidural anesthesia for cesarean section, L2-L3 interspace	20 mL, L3-L4, POD 2 20 mL, L2-L3, POD 5	Radicular pain in back, buttocks, and posterior aspect of the lower extremities, POD 5	Subdural hematoma from L4-L5 and another at L5	Partial resolution of symptoms during the following months

PPD = postpartum day, POD = postoperative day.

## Abbreviations

EBP: Epidural blood patch
PDPH: Postdural puncture headache
MRI: Magnetic resonance imaging
PPD: Postpartum day
POD: Postoperative day
BMI: Body mass index
LOR: Loss of resistance
CT: Computed tomography.
